# Unhealthy snack intake modifies the association between screen-based sedentary time and metabolic syndrome in Brazilian adolescents

**DOI:** 10.1186/s12966-019-0880-8

**Published:** 2019-11-27

**Authors:** Camila Wohlgemuth Schaan, Felipe Vogt Cureau, Deborah Salvo, Harold W. Kohl, Beatriz D. Schaan

**Affiliations:** 1 Post-graduate Program in Endocrinology, Universidade Federal do Rio Grande do Sul, Porto Alegre. Hospital de Clínicas de Porto Alegre, St. Ramiro Barcelos 2350/21, 90035-003, Porto Alegre, RS Brazil; 2 Washington University in St. Louis, Brown School, Prevention Research Center in St. Louis, Saint Louis, MO USA; 3 University of Texas Health Science Center at Houston, School of Public Health, Michael and Susan Dell Center for Healthy Living and University of Texas at Austin, Department of Kinesiology and Health Education, Austin, TX USA

**Keywords:** Adolescents, Metabolic syndrome, Screen time

## Abstract

**Background:**

Excessive screen time has been associated with metabolic syndrome (MetS) among adolescents; however, snack intake in front of screens may play a role in this association. Therefore, our objective was to investigate the association between screen-based sedentary time with MetS and whether this association is modified by unhealthy snack intake in front of screens.

**Methods:**

This study was a nationwide, cross-sectional, school-based survey in Brazil including adolescents aged 12 to 17 years. The frequency of snack consumption in front of screen and screen-based sedentary time (TV view, computers and videogames use) were self-reported. Thereafter, screen time was categorized (≤2, 3–5 and ≥ 6 h/day); snack consumption in front of screens was dichotomized. Metabolic syndrome diagnosis was defined based on the International Diabetes Federation criteria. Associations between screen time and MetS were investigated using logistic regression in overall sample and after stratification by snack intake in front of screens.

**Results:**

A total of 33,900 adolescents were included in the analysis. The final adjusted model, which included sociodemographic data, physical activity, and energy intake, showed that adolescents who spent ≥6 h/day in front of screens had an increased odds ratio for MetS (OR = 1.68, 95%CI: 1.03–2.74). However, after stratifying the sample according to reported snack intake, the association between higher screen-based sedentary time and MetS remained significant only for adolescents who reported consumption of snacks in front of screens.

**Conclusion:**

Longer screen-based sedentary times were directly associated with MetS. However, this association seems to be modified by reported snack intake in front of screens.

## Introduction

Despite its decline in the past few years, cardiovascular disease remains the main cause of mortality in Brazil, accounting for 31% of all deaths [1]. Cardiovascular risk factors in youth, such as obesity, hypertension, dyslipidemia, and hyperglycemia, are known predictors of cardiovascular disease [2]. The co-occurrence of these typical cardiovascular risk factors, defined as the metabolic syndrome (MetS), reached a prevalence of 2.6% among the general Brazilian adolescent population, while affecting one in every five youths with obesity [3]. Additionally, MetS in adolescence presents a higher risk for the development of subclinical atherosclerosis and type 2 diabetes mellitus during adulthood [4].

Previous studies have shown that certain unhealthy behaviors are associated with a higher prevalence of MetS. Usually, these behaviors include short sleep duration [5], physical inactivity [6], and unhealthy diet [7]. More recently, excessive sedentary time, defined as activities with low energy expenditure (≤1.5 METs) in a sitting or reclining position, including screen-based entertainment [8], has been linked with poor cardio-metabolic health [9]. Due to the negative impact of excessive screen-based entertainment, the American Academy of Pediatrics recommends that children and adolescents limit it to no more than two hours per day [10].

The exact mechanisms involved in the possible association between screen-based sedentary time and MetS remain unclear. The detrimental effects of screen time were previously attributed to displaced time spent in physical activity (i.e., of moderate to vigorous intensity). Screen-based sedentary time has also been consistently associated with unhealthy eating habits, such as a high intake of soft drinks and unhealthy snacks, which have been proven to be also related to obesity and MetS [11]. Moreover, a previous study showed that TV viewing time and unhealthy snack food consumption are jointly associated with MetS and its components [12].

In adolescents, the detrimental association between screen-based sedentary time and MetS seems to be independent of overall physical activity [9] and dietary intake [13]. However, the influence of the frequency of unhealthy snacking, as a food behavior, specifically during screen time is not yet completely established. Thus, our objective was to investigate the association between screen-based sedentary time with MetS and whether this association is modified by unhealthy snack intake in front of screens in a large sample of Brazilian adolescents.

## Methods

### Design and sample

The Study of Cardiovascular Risks in Adolescents (Estudo de Riscos Cardiovasculares em Adolescentes, “ERICA”) was a multicenter, nationwide, cross-sectional, school-based study carried out in urban and rural settings in Brazil. The sample was composed of students between 12 and 17 years of age enrolled in private and public schools in Brazilian municipalities with a minimum population of 100,000 inhabitants. According to the most recent Brazilian census, more than half of the Brazil’s population (57%) lives in these municipalities [14]. Data were collected between February 2013 and November 2014.

For sampling purposes, the target population was divided into 32 geographic strata: all 26 State capitals, the Federal District, and five more strata representing other municipalities with at least 100,000 inhabitants in each region of Brazil. The schools were selected based on probability proportional to size (number of students) and inversely proportional to the distance between the school municipality and the state capital. In total, 1247 schools in 124 municipalities were selected. Three classrooms, with different combinations of scheduled time at school (morning and afternoon) and grade (seventh, eighth and ninth grade of Elementary and first, second and third grade of High School), were randomly selected from each school, and all students in these classes were invited to participate in ERICA [15].

For this study, we used data from students who attended school during the morning, including students in the integral or semi-integral system, as overnight fasting was mandatory for the blood sampling. We excluded adolescents outside the age range of 12–17 years, pregnant girls, those with any type of disability and who did not provide data for the main variables (screen time and snack intake) or did not participated in blood sampling. Thus, the total available sample size for these analyses was 33,900 adolescents. Further details regarding the sampling, design and participation in the ERICA can be found in previous publications [15–17].

ERICA was approved by the Research Ethic Committee at the Institute for Studies in Public Health of Universidade Federal do Rio de Janeiro (ERICA’s central Committee) and in Research Ethics Committees at all other 26 Federation units in Brazil. All participants and their legal guardians provided written consent to participate in the study.

### Metabolic syndrome and its components

Waist circumference (WC) was included as a marker of central adiposity. Measurements were performed twice at a point midway between the iliac crest and the lower costal margin. Blood pressure was assessed using a digital monitor (Omron 705-IT) previously validated for use in youths [18]. Blood pressure was taken from each student’s right arm using individual cuff sizes after five minutes of sitting still. Three consecutive measurements for each individual were performed with an interval of at least three minutes between them and the mean of the last two measurements was considered for analysis [15].

All participants were asked to refrain from eating for at least 10–12 h before the blood sampling. Compliance with the overnight fast was determined by a questionnaire before venipuncture. Fasting blood samples were collected for measuring glucose, high-density lipoprotein cholesterol (HDL-c) and triglycerides. All blood samples were analyzed by a single laboratory following a standardized protocol [19].

Metabolic syndrome was defined according to the International Diabetes Federation criteria [20]. These include a high WC as a mandatory component (< 16 years: ≥ 90th percentile; ≥ 16 years, males: ≥ 90 cm; and ≥ 16 years, females: ≥ 80 cm) and at least two of the following criteria: [1] fasting glucose ≥100 mg/dl [2]; systolic blood pressure ≥ 130 mmHg or diastolic blood pressure ≥ 85 mmHg [3]; triglycerides ≥150 mg/dl [4]; HDL-c in adolescents < 16 years: < 40 mg/dl; HDL-c in males ≥16 years: < 40 mg/dl; and HDL-c in girls > 16 years: < 50 mg/dl.

### Screen-based sedentary time

Time spent in front of a screen was assessed using a single question: during an ordinary weekday, how many hours do you spend watching television, using the computer, or playing videogames? This question was previously validated and showed moderate to high accuracy to categorize those adolescents reporting more than two hours/day in front of screens [21].

Considering that a large proportion of Brazilian adolescents do not limit screen time to at least two hours per day [22], we adopted a categorization for screen time that allowed us to examine a potential dose–response association with MetS. The first category represents the adolescents who met the recommendations for limit screen time (≤ 2 h/day), followed by those who are considered “sedentary” (3–5 h/day), whereas the last category (≥ 6 h/day) identified those who are ‘highly sedentary’ and may have additional health risk compared with the second category.

### Snacking in front of screens

The participants also reported their habits regarding the snacks intake (i.e., popcorn, cookies, pretzels, sandwiches, chocolate, or candies) in two different situations: [1] in front of the TV and [2] in front of the computer/videogame. Thereafter, we have combined both these variables to investigate the general snack intake in front of screens. For analysis purposes, these variables were dichotomized into “no” (if the participant reported “never” eat snacks in front of a screen) or “yes” (if they reported eating snacks sometimes, almost every day or every day).

### Covariates

Covariates considered in this study included: sex, age (12 to 17 years), region of the country (North, Northeast, Midwest, Southeast, and South), and self-reported skin color (white, black, brown, and yellow/indigenous). Socioeconomic status was assessed with an questionnaire used in the Brazilian Demographic Census [23], which takes into account possession of specific material goods and the presence of a housekeeper at home (ranging from 0 to 38 points).

Physical activity was assessed using an adapted version of the Self-Administered Physical Activity Checklist [24]. This instrument consisted in a list of 24 activities (leisure time and commuting) and allowed the adolescents to report the frequency (days) and duration (hours and minutes) of these activities during the last seven days. To determine the weekly amount of time spent in physical activity, we multiplied self-reported duration (min) and frequency (times/week) for each reported activity and summed all of them. This questionnaire has been validated in Brazilian adolescents [25].

Total energy intake (kcal/day) was estimated using a face-to-face 24 h food recall interview performed by trained interviewers using the multiple-pass interview technique, aiming at reducing potential under-reporting of food consumption [15]. The ERICA’s 24 h food recall was specifically developed for the study and uses a database composed of 1626 food items [26]. Moreover, to estimate intake of energy and nutrients, ERICA considered the Brazilian Food Composition Table [27] and the Brazilian Portion Size Table [28]. All information provided was detailed regarding the method of preparation and quantity. Pictures included in the software were used to facilitate the estimation of the portion size consumed [26].

### Statistical analysis

To obtain population-representative estimates, we used sampling weights calculated for ERICA, which account for the complex survey design [17]. Descriptive statistics were used to calculate means or proportions and their 95% confidence intervals (95% CI). The associations between screen time categories (≤ 2 h/day, 3 to 5 h/day, and ≥ 6 h/day) and MetS were calculated using logistic regression models to estimate the odds of MetS, given each level of screen time. The lowest screen-time duration category (≤ 2 h/day) was the reference group.

Multivariable analysis, with models adjusting for potential confounding variables, were conducted in four modeling levels, as follows: sex and age (basic model); in the second model, we added socioeconomic level, skin color, and region (full sociodemographic model); in the third model, we added total energy intake (full sociodemographic + total energy intake model); finally, physical activity was included (full sociodemographic + total energy intake in kcal/day + physical activity in min/week - model). All potential confounders were selected based on the literature and they were kept in the model after the variables entry. The global adjustment of the models was tested for goodness of fit using Pearson’s test. We did not find evidence of multicollinearity in the analysis.

In further analysis, we tested the statistical interaction between screen time and snack intake in front of screens in relation to MetS. It was examined using a multiplicative approach through the Wald’s test for heterogeneity. Three interaction terms were built [ [1] screen time X snack in front of TV [2]; screen time X snack in front of computer/video game [3]; screen time X snack in front of all screens] and included in the model apart. The *p*-values of Wald’s test for interaction were 0.001 for snack intake in front of the TV, 0.464 for snack intake in front of the computer/videogame and 0.007 for snack in front of all screens.

Data were analyzed using the Stata software package (College Station, TX, USA), version 14 and considering the “*svy*” prefix command. All tests were two-tailed, and a significance of 5% for type I error was adopted in all analysis.

## Results

Overall, 36,956 adolescents participated in ERICA from which 33,900 adolescents provided complete information on main variables. In total, 3056 adolescents were excluded from the analyses because they either did not report or did not remember information about screen time. Greater proportions of girls, younger adolescents, people from low-income families, and with black skin color, as well as a lower proportion of physically inactivity adolescents were not included due to missing data (Additional file 1).

The majority of the sample was composed of females (59.4%) and mean age was 14.6 years (95% CI: 14.6–14.7). Almost half of the sample reported having brown skin color (49.2%) and 37.8% of the adolescents were classified as physically active. Mean time of physical activity in this sample was 683 min/wk. (IC95%: 659–708). The majority of the adolescents reported eating snacks in front of screens. Overall, 40.5% of the adolescents limit screen time to no more than two hours per day and the prevalence of MetS was 2.6% (95% CI: 2.3–3.0). With the exception of high blood glucose, the prevalence of all other MetS components was higher in the highest screen time category (Table 1).

**Table 1:** Characteristics of the whole sample and classified by screen time categories (*n* = 33,900). ERICA 2013/2014

	All sample (*n* = 33,900)	Screen time categories		
		≤ 2 h/day (*n* = 14,526)	3 to 5 h/day (*n* = 12,902)	≥ 6 h/day (*n* = 6,472)
	% (95% CI)			
Overall		40.5 (38.6–42.3)	39.0 (37.5–40.6)	20.5 (19.5–21.5)
Sex^a^				
Female	59.4 (58.9–60.0)	60.4 (59.6–61.2)	58.6 (57.8–59.5)	59.0 (58.7–60.2)
Male	40.6 (40.0–41.1)	39.6 (38.8–40.4)	41.4 (40.5–42.2)	41.0 (39.8–42.2)
Age group^a^, years				
12–13	27.7 (27.2–28.2)	29.4 (28.6–30.1)	25.9 (25.2–26.7)	27.3 (26.3–28.4)
14–15	36.3 (35.7–36.8)	33.9 (33.2–34.7)	37.3 (36.4–38.1)	39.4 (38.2–40.6)
16–17	36.1 (35.6–36.6)	36.7 (35.9–37.5)	36.8 (35.9–37.6)	33.2 (32.1–34.4)
Socioeconomic level, tertiles				
1st (lowest)	35.1 (33.3–37.0)	38.2 (35.9–40.7)	32.4 (30.0–35.0)	34.1 (31.1–37.2)
2nd	34.2 (33.1–35.3)	34.0 (32.0–36.2)	33.8 (31.7–36.0)	35.2 (32.7–37.7)
3rd	30.7 (28.7–32.8)	27.7 (25.1–30.5)	33.8 (31.0–36.7)	30.7 (27.4–34.3)
Skin color				
White	41.1 (39.2–43.1)	39.3 (37.0–41.7)	41.9 (39.4–44.5)	43.1 (39.5–46.8)
Black	7.1 (6.3–8.0)	7.6 (6.4–9.0)	6.6 (5.5–7.9)	7.2 (6.1–8.5)
Brown	49.2 (47.3–51.1)	50.7 (48.5–52.8)	49.0 (46.3–51.6)	46.8 (43.6–50.1)
Yellow/indigenous	2.5 (2.1–3.1)	2.4 (1.9–3.0)	2.6 (2.0–3.3)	2.8 (2.1–3.8)
Total energy intake, kcal	2312 (2249–2376)	2275 (2167–2384)	2319 (2277–2362)	2372 (2289–2455)
Active (≥ 420 min/week)	37.8 (36.7–38.8)	39.3 (37.5–41.1)	38.3 (36.7–40.0)	33.8 (30.8–36.8)
unhealthy snacks intake in front of TV				
No	14.8 (14.0–15.7)	19.9 (18.4–21.5)	12.4 (11.0–14.0)	9.5 (7.7–11.7)
Yes	85.2 (84.3–86.0)	80.1 (78.5–81.6)	87.6 (86.0–89.0)	90.5 (88.3–92.3)
unhealthy snacks intake in front of PC/video game				
No	36.2 (34.5–37.9)	51.5 (49.7–53.3)	28.6 (26.4–31.0)	20.3 (18.1–22.7)
Yes	63.8 (62.1–65.5)	48.5 (46.7–50.3)	71.4 (69.0–73.6)	79.7 (77.3–81.9)
unhealthy snacks intake in front of all screens				
No	10.2 (9.4–11.2)	15.7 (14.3–17.3)	7.3 (6.0–8.9)	5.0 (3.5–7.0)
Yes	89.8 (88.8–90.6)	84.3 (82.7–85.7)	92.7 (91.1–94.0)	95.0 (93.0–96.5)
Components of MetS^b^				
High waist circumference	12.7 (11.6–13.9)	11.4 (10.2–12.6)	13.4 (11.7–15.3)	14.0 (12.0–16.4)
High triglycerides	4.5 (4.0–5.1)	4.2 (3.7–4.8)	4.4 (3.7–5.2)	5.4 (4.1–7.0)
High blood glucose	4.0 (3.5–4.7)	4.2 (3.3–5.3)	4.4 (3.5–5.5)	3.0 (2.4–3.8)
High blood pressure	8.3 (7.7–9.0)	7.6 (6.7–8.6)	8.4 (7.5–9.3)	9.4 (7.5–11.7)
Low HDL – c	32.5 (30.0–35.1)	32.5 (29.9–35.3)	32.0 (29.0–35.1)	33.6 (29.9–37.4)

*CI* confidence interval, *HDL* High density lipoprotein, *MetS* metabolic syndrome

^a^ Non-weighted values

^b^The components of MetS were classified according sex and age-specific recommendations of the International Diabetes Federation

**Fig. 1:**
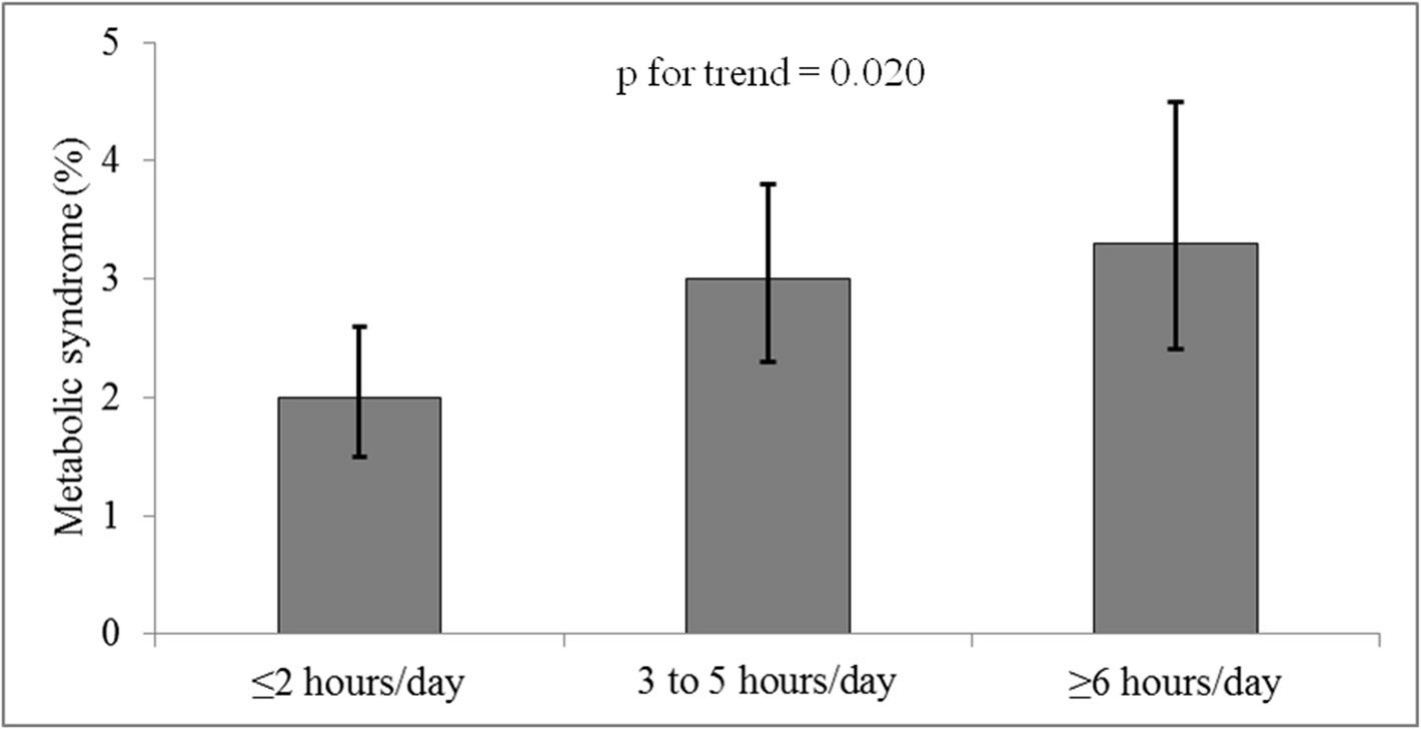
Prevalence of metabolic syndrome (MetS) according to the screen time categories among Brazilian adolescents. Metabolic syndrome was classified according to sex and age-specific recommendations of the International Diabetes Federation

The prevalence of MetS across the screen time categories is shown in Fig. 1. The prevalence of MetS was 1.9% (95% CI: 1.5–2.5), 3.0% (95% CI: 2.3–3.8), and 3.3% (95% CI: 2.4–4.4) among adolescents who spent ≤2 h/day, 3–5 h/day, and ≥ 6 h/day in front of screens, respectively (Fig. 1). The *p*-value for trends in the association between screen time and MetS was 0.02.

In the logistic regression models, we observed a dose-response gradient for the association between screen time categories and MetS (Table 2). However, the 95% CI for the intermediate category (3–5 h/day) overlapped with unity after the adjustment for selected covariates. In the final adjusted model, the odds ratio for MetS was 1.39 (95% CI: 0.96–2.02) and 1.68 (95% CI: 1.03–2.74) among adolescents who spent 3–5 h/day and ≥ 6 h/day in front of screens, respectively.

**Table 2:** Association between screen time categories and MetS among Brazilian adolescents. ERICA 2013/2014

	Screen time categories		
	≤ 2 h/day	3 to 5 h/day	≥ 6 h/day
	OR (95%CI)		
Unadjusted model			*p for trend = 0.020*
	1	1.55 (1.07–2.24)	1.69 (1.04–2.75)
Model 1: adjusted by sex and age			*p for trend = 0.023*
	1	1.52 (1.05–2.22)	1.70 (1.04–2.78)
Model 2: adjusted by model 1 + skin color, socioeconomic level and region			*p for trend = 0.041*
	1	1.39 (0.95–2.02)	1.66 (1.01–2.76)
Model 3: adjusted by model 2 + energy intake (kcal)			*p for trend = 0.031*
	1	1.40 (0.97–2.04)	1.70 (1.03–2.80)
Model 4: adjusted by model 3 + physical activity			*p for trend = 0.033*
	1	1.40 (0.97–2.04)	1.70 (1.03–2.82)

*OR* odds ratio, *CI* confidence interval, *MetS* metabolic syndrome

**Fig. 2:**
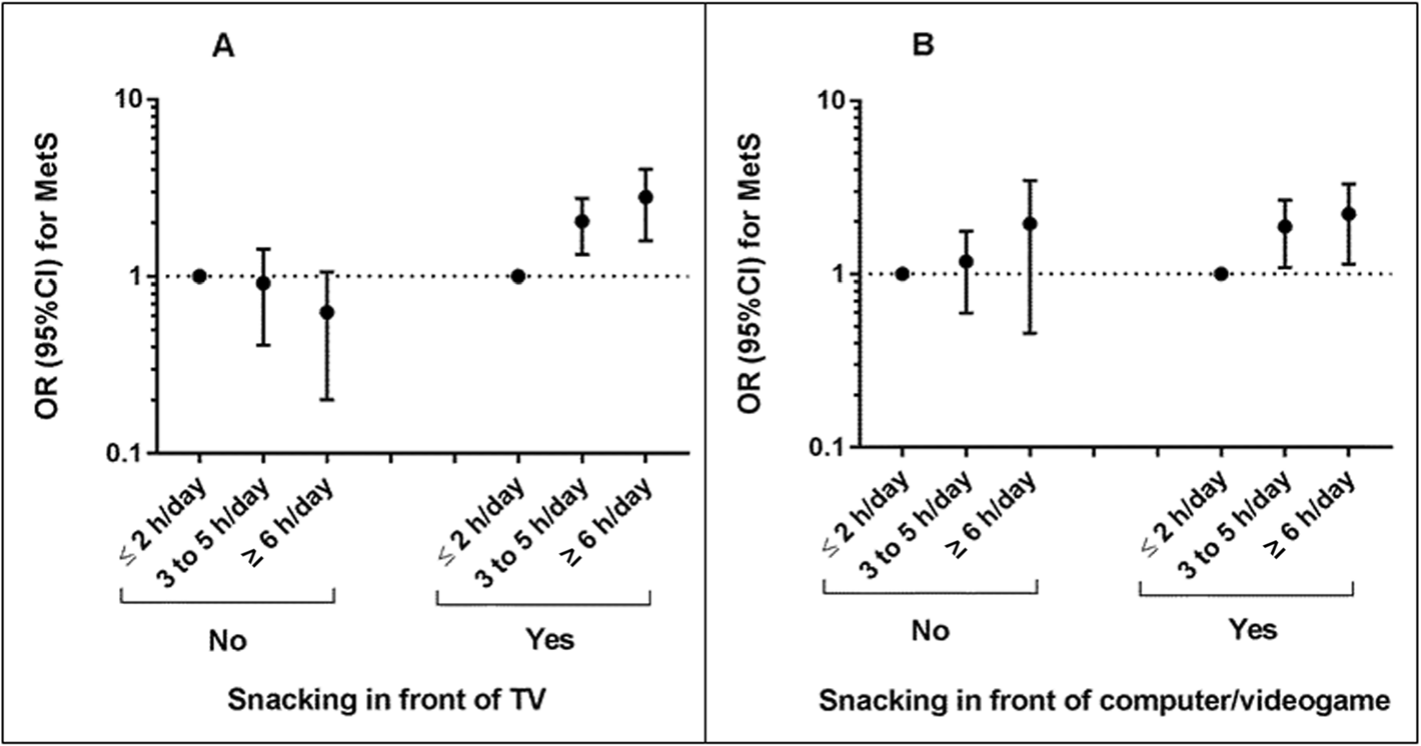
Association between screen time categories and metabolic syndrome (MetS): (**a**) effect of screen time on MetS, by unhealthy snacking in front of TV and (**b**) effect of screen time on MetS, by unhealthy snacking in front of computer/videogame. OR: odds ratio; CI: confidence interval; h/day: hours per day; adjusted by sex, aged, skin color, socioeconomic level, region, energy intake (kcal) and physical activity. Footnote: Panel A - NO: OR: 0.90, 95% CI: 0.42–1.93 for 3 to 5 h/day; OR: 0.53, 95% CI: 0.26–1.10 for ≥6 h/day. YES: OR: 1.96, 95% CI: 1.37–2.80 for 3 to 5 h/day; OR: 2.63, 95% CI: 1.68–4.11 for ≥6 h/day. Panel B – NO: OR: 1.09, 95% CI: 0.65–1.81 for 3 to 5 h/day; OR: 1.57, 95% CI 0.69–3.61 for ≥6 h/day. YES: OR: 1.77, 95%CI: 1.15–2.72 for 3 to 5 h/day; OR: 2.05, 95% CI: 1.24–3.38 for ≥6 h/day.

Considering the interaction observed between screen time and snack intake categories, we performed stratified analysis (Fig. 2). After stratification by reported snack intake, there was no significant association between screen time and MetS among adolescents who reported no snacking in front of screens. Meanwhile, the odds ratio for MetS was gradually higher through screen time categories, among those who reported habitual consumption of snacks in front of the TV, computers/videogames or both (OR: 2.09, 95% CI: 1.33–3.26 and OR: 2.50, 95% CI: 1.46–4.27 for 3 to 5 h/day and ≥ 6 h/day, respectively).

## Discussion

Our results suggest that screen time is associated with MetS in Brazilian adolescents, even after controlling for potential confounding factors, including total energy intake and physical activity. However, the association between screen time and MetS in this population appeared to be modified by the unhealthy snacking behavior in front of screens, remaining significantly associated only among those adolescents who have reported habitual unhealthy snacking in front of screens.

We observed that the prevalence of MetS components, with exception of high blood glucose, increased slightly through the screen time categories. This is consistent with findings from other studies on young subjects that reported a positive association between screen time and adiposity [29] (a main component of MetS) and with other cardio-metabolic risk factors, such as high blood pressure and high total cholesterol [30]. Sedentary behavior results in increased insulin resistance and lower skeletal muscle myofibrillar protein synthesis [31]. Moreover, sedentary time leads to longer periods of postprandial hyperglycemia [32]. However, perhaps due to the low prevalence of high blood glucose, especially in the highest screen time category, it was not observed in this study as we observed in the other components of the MetS.

Some studies have demonstrated that screen time is associated with MetS in youth, with possible long-term repercussions in adulthood [9, 33]. For example, studies in representative samples of both American [9] and Korean [34] adolescents observed an odds ratio twice as high among adolescents who spent at least five hours of screen time per day, when compared with those adolescents who reported less screen time. In this context, we believe that our study adds to previous findings by showing that total screen time, in addition to TV watching, was independently associated with MetS in a large sample of adolescents from a developing country and that unhealthy snacking in front of screens may play a role in the observed association.

In relation to the snacking habits and screen time, Hobbs et al. observed that TV viewing and total screen time were associated with elements of an unhealthy diet among adolescents [35]. However, the authors highlight the need to interpret the associations carefully due to the high heterogeneity and scarcity of studies that explore the relationship between sedentary time and dietary behaviors, which limits the available evidence [35]. We extend these previous observations, showing that the unhealthy snack intake in front of screens can modify the association between screen time and MetS. In this context, prospective studies among adolescents are required to better understand this issue.

Additionally, Collings et al. [36] showed no evidence of associations between eating while watching TV or the presence of a TV in a child’s bedroom with adiposity markers. However, their sample was composed by young children (3-year-olds), and it is plausible that these associations may not be present during the preschool years due to less autonomy of dietary choices at this age. Other data (in US adolescents), suggest that TV viewing is positively associated with MetS and, after examining the mediating effects, sugar-sweetened beverages and fruit and vegetable consumption showed partial mediation effects in the reported association [13].

Our findings suggest that excessive screen time combined with habitual unhealthy snacking in front of screens can be particularly harmful for metabolic health among adolescents. We hypothesized that behavioral relations between screen time and unhealthy snacking are connected; however the behavioral mechanism involved remains unclear. It is possible that adolescents who spent more time in front of screens are more exposed to advertisements of fast-food, sugar-sweetened beverages, and ultra-processed foods [36]. Also, unhealthy snacks, such as finger foods, are more practical to be consumed in front of screens [37].

Thus, the causal direction of these associations remains to be determined. It is possible that more unhealthy snacking in front of a screen is the direct result of more exposure to the screens. A circular pattern could also be occurring, i.e., those who tend to snack while in front of a screen also tend to spend more total screen-time, and this in turn leads to even more snacking. Future longitudinal study designs will be critical to truly understand the interplay of these complex relations, and as such, can help develop recommendations to implement intervention strategies to promote healthy behavior among adolescents. Unhealthy snacking while watching TV could also simulate “mindless eating”, which in this case is defined as ignoring the physiological signs of fullness and relying on external cues (such as the end of the TV show) to signal normal meal satiety [38].

Our results have some implications for public health. Excessive screen time is associated with higher odds of MetS in adolescents, even after adjusting for physical activity or total energy intake. However, we observed that consumption of snacks in front of screens may modify this association, playing a role in that issue. Thus, prevention programs aimed at reducing screen-based sedentary time and the unhealthy snack intake in front of screens should be developed in combination [39]. Screens are ubiquitous and young people live surrounded by screens, so to limit food consumption in front of screens can be more suitable than avoid screen based activities. Also, it is important that the Brazilian government implement policies and regulations to restrict the advertisement of high-fat, high-salt, and high-sugar foods on children’s channels and in-between children’s shows, following the example of other countries [40]. However, this may be only one of the many polices needed to combat obesity and cardiometabolic risks among adolescents.

### Strengths and limitations

Our study has important strengths. We used a country-wide representative sample of Brazilian adolescents, which allowed us to stratify the analysis based on snack consumption, and produce results using sampling weights, and thus are generalizable to Brazilian adolescents at large. In addition, we considered many potentially confounding factors, including physical activity and energy intake. Finally, data collection was standardized, and all biomarkers were analyzed in the same central laboratory. We also used a standardized definition of MetS and screen-based sedentary time categories, which allowed us to explore dose-response associations. Moreover, this study is the first of its kind in Brazil and, to our knowledge, there are few previous studies which have explored the interaction between excessive screen time and snack consumption in relation to the occurrence of MetS among adolescents.

However, this study has some limitations too. Firstly, as a cross-sectional study, it did not allow for establishing causal relationships. Secondly, screen time and snack consumption were assessed by self-report questionnaires. Moreover, questions used to assess snacking in front of screens were not fully validated, which may introduce reporting bias. However, non-differential measurement error attenuates, rather than increases, the apparent magnitude of associations, and as such we can speculate that the apparent effects would be much greater with a more accurate measurement (i.e., direct measurement or the combination of an accelerometer and a recall). Because of the large sample size it was difficult to use objective measures such as accelerometers in studies like ERICA. Finally, screen time was assessed only for weekdays and the question used did not allowed us to investigate different domains of screen time.

Although screen time measures in youth have acceptable test–retest reliability, their validity remains unknown. Finally, due to the low prevalence of MetS, the statistical strength of this study may be insufficient to detect subtle associations after split the sample for snack consumption. Although we did adjust the models for important confounding factors, the residual confounding factors and the role of snacks as mediators remains a possibility.

## Conclusion

High screen-based sedentary time is positively associated with MetS, but this association remains significant only among adolescents who report unhealthy snacking in front of screens. Our results suggest that strategies to address MetS in the adolescent population should aim at limiting unhealthy snacking while in front of a screen. This strategy may reduce the strength of the association between total screen time and MetS among adolescents.

## Supplementary information

**Additional file 1.** Characteristics of the sample included vs. not included.

## Data Availability

The datasets generated and/or analyzed during the current study are not publicly available due to rules of ERICA project.
