# Supplementary material for: Unhealthy snack intake modifies the association between screen-based sedentary time and metabolic syndrome in Brazilian adolescents
Source: Int J Behav Nutr Phys Act. 2019 Nov 27;16:115. doi: 10.1186/s12966-019-0880-8 (PMC6882160; doi:10.1186/s12966-019-0880-8)
Supplement: Supplementary file 1 — Additional file 1. Characteristics of the sample included vs. not included. [file 12966_2019_880_MOESM1_ESM.docx]

Characteristics of the sample included *vs*. not included

|  | Included (n=33,900) | Not included  (n=3,056) |
| --- | --- | --- |
| Female sex, % (95%CI) | 49.5 (49.1-49.9) | 57.6 (53.0-62.0) |
| Age (years), mean (95%CI) | 14.63 (14.62-14.65) | 14.37 (14.21-14.52) |
| Socioeconomic level tertile % (95%CI) |  |  |
| 1 (lowest) | 35.0 (33.1-36.8) | 41.9 (37.3-46.7) |
| 2 | 34.2 (33.1-35.4) | 32.7 (29.0-36.7) |
| 3 | 30.8 (28.8-32.9) | 25.4 (21.2-30.0) |
| Skin Color % (95%CI) |  |  |
| White | 41.2 (39.3-43.3) | 32.6 (28.3-37.3) |
| Black | 7.1 (6.3-8.0) | 16.8 (12.7-21.8) |
| Brown | 49.1 (47.2-51.0) | 47.5 (43.0-52.0) |
| Yellow/indigenous | 2.5 (2.1-3.0) | 3.1 (1.6-6.1) |
| Total energy intake (kcal), mean (95%CI) | 2315 (2251-2380) | 2073 (1968-2177) |
| MVPA (min/day), median (95%CI) | 50 (47.8-52.1) | 34.3 (27.2-41.2) |
| Snacks intake in front of TV % (95%CI) |  |  |
| No | 14.9 (14.0-15.8) | 16.3 (12.6-21.0) |
| Yes | 85.0 (84.2-86.0) | 83.6 (79.0-87.4) |
| Snacks intake in front of computer % (95%CI) |  |  |
| No | 36.0 (34.3-37.8) | 39.2 (35.2-43.4) |
| Yes | 64.0 (62.2-65.7) | 60.7 (56.5-64.8) |

CI: confidence interval; MVPA: moderate to vigouros physical activity/ TV: television.
